# Evidence for cadherin-11 cleavage in the synovium and partial characterization of its mechanism

**DOI:** 10.1186/s13075-015-0647-9

**Published:** 2015-05-15

**Authors:** Erika H Noss, Gerald FM Watts, Davide Zocco, Tracy L Keller, Malcolm Whitman, Carl P Blobel, David M Lee, Michael B Brenner

**Affiliations:** Division of Rheumatology, Immunology, and Allergy, Brigham and Women’s Hospital and Harvard School of Medicine, Smith Research Building, 5th floor, 1 Jimmy Fund Way, Boston, MA 02115 USA; Exosomics Siena S.p.A., Strada del Petriccio e Belriguardo, 35, 53100 Siena, Italy; Harvard School of Dental Medicine, Department of Developmental Biology, REB 505, 190 Longwood Avenue, Boston, MA 02115 USA; Hospital for Special Surgery, 535 east 70th Street, New York, NY 10021 USA; F. Hoffman-La Roche Ltd, Grenzacherstrasse 124, Building 69/Room 206, 4070, Basel, Switzerland

## Abstract

**Introduction:**

Engagement of the homotypic cell-to-cell adhesion molecule cadherin-11 on rheumatoid arthritis (RA) synovial fibroblasts with a chimeric molecule containing the cadherin-11 extracellular binding domain stimulated cytokine, chemokine, and matrix metalloproteinases (MMP) release, implicating cadherin-11 signaling in RA pathogenesis. The objective of this study was to determine if cadherin-11 extracellular domain fragments are found inside the joint and if a physiologic synovial fibroblast cleavage pathway releases those fragments.

**Methods:**

Cadherin-11 cleavage fragments were detected by western blot in cell media or lysates. Cleavage was interrupted using chemical inhibitors or short-interfering RNA (siRNA) gene silencing. The amount of cadherin-11 fragments in synovial fluid was measured by western blot and ELISA.

**Results:**

Soluble cadherin-11 extracellular fragments were detected in human synovial fluid at significantly higher levels in RA samples compared to osteoarthritis (OA) samples. A cadherin-11 N-terminal extracellular binding domain fragment was shed from synovial fibroblasts after ionomycin stimulation, followed by presenilin 1 (PSN1)-dependent regulated intramembrane proteolysis of the retained membrane-bound C-terminal fragments. In addition to ionomycin-induced calcium flux, tumor necrosis factor (TNF)-α also stimulated cleavage in both two- and three-dimensional fibroblast cultures. Although cadherin-11 extracellular domains were shed by a disintegrin and metalloproteinase (ADAM) 10 in several cell types, a novel ADAM- and metalloproteinase-independent activity mediated shedding in primary human fibroblasts.

**Conclusions:**

Cadherin-11 undergoes ectodomain shedding followed by regulated intramembrane proteolysis in synovial fibroblasts, triggered by a novel sheddase that generates extracelluar cadherin-11 fragments. Cadherin-11 fragments were enriched in RA synovial fluid, suggesting they may be a marker of synovial burden and may function to modify cadherin-11 interactions between synovial fibroblasts.

**Electronic supplementary material:**

The online version of this article (doi:10.1186/s13075-015-0647-9) contains supplementary material, which is available to authorized users.

## Introduction

Synovial fibroblasts are joint stromal cells with important roles in the normal and inflammatory synovium [1, 2]. In the normal joint, fibroblasts remodel connective tissue matrix and secrete the synovial fluid lubricants hyaluronan and lubricin. In the inflamed joint, fibroblast hyperplasia contributes to pannus development, and fibroblast activation produces many mediators that promote inflammation, cartilage erosion, angiogenesis, and bone erosion.

Understanding the role of synovial fibroblasts in the joint has been advanced by the discovery that the cell adhesion molecule cadherin-11 specifically regulates synovial morphogenesis and synovial fibroblast function [3–8]. Cadherin-11 belongs to the cadherin family of cell-to-cell adhesion molecules that mediate homophilic adhesion, namely a cadherin of one type binds to cadherin of the same type in *trans* on a neighboring cell through interactions between their extracellular domains [9]. Cadherins have well-described roles in organ morphogenesis and tissue homeostasis. In the joint, cadherin-11 is critical for synovial development. Mice genetically ablated for cadherin-11 develop a hypoplastic synovium and, when challenged in an inflammatory arthritis model, show both reduced inflammation and cartilage erosion, providing direct *in vivo* evidence for the function of cadherin 11 and fibroblasts in the normal and inflamed synovium [6].

However, cadherins do not just passively mediate cell adhesion. Through interactions with catenins and other signaling molecules at their cytoplasmic domain, cadherins actively alter cell signaling pathways [10, 11]. In synovial fibroblasts, cell surface cadherin-11 engagement with a recombinant soluble form of the cadherin-11 extracellular binding domain linked to immunoglobulin Fc tail induced mitogen activated protein kinase and nuclear factor-κB activation, leading to marked IL-6, chemokine, and metalloproteinase expression [3, 7]. Furthermore, cadherin-11 signaling acted synergistically with inflammatory cytokines (for example, TNF-α) to amplify expression of these inflammatory and degradative mediators. These results suggest a model where increased number and/or turnover of cadherin-11 complexes may help to directly promote fibroblast activation in the synovium under inflammatory conditions in rheumatoid arthritis (RA).

Cadherin turnover occurs dominantly through endosomal internalization followed by either recycling back to the cell surface or degradation in the lysosomes [12, 13]. However, an additional pathway for cadherin turnover has been described. Some cadherins undergo stepwise cleavage from the cell membrane by ectodomain shedding followed by regulated intramembrane proteolysis, a cleavage process with over 90 described protein substrates [14, 15]. In this pathway, the first cleavage releases the protein ectodomain and is mediated by a variety of cell sheddases, mainly a disintegrin and metalloproteinase (ADAM) family members, with the aspartyl proteases beta-secretase (BACE)1 and BACE2 having a more limited number of substrates. The remaining membrane stub can be further cleaved in the transmembrane domain to release a free cytosolic intracellular domain, a process known as regulated intramembrane proteolysis. This cleavage occurs by a limited number of intramembrane-cleaving proteases (I-CLiPs). Type I proteins, like cadherins, are almost exclusively cleaved by γ-secretase, a multiprotein complex containing the GXGD-type aspartyl protease presenilin [16].

The biologic consequences of this regulated cleavage pathway are diverse [14, 16]. Depending on the membrane protein, cleavage may act to terminate signals from engaged receptors or change the cell adhesive state. In addition, for many molecules, the cleavage fragments have important functions. For example, soluble ectodomains may promote paracrine cell signaling, as seen with epidermal growth factor ligand cleavage, while intracellular domains may act as transcription factors, as seen with Notch activation.

Cadherin cleavage may also have diverse functions, likely depending on the cadherin, cell, and stimuli that induce cleavage. Cadherin cleavage may help regulate the levels of cell-to-cell contacts. For example, induction of E-cadherin cleavage in tumor cells leads to dramatic disassembly of cell-to-cell contacts, while blockade of cleavage through genetic ablation of ADAM10 increases N-cadherin surface levels on mouse fibroblasts [17, 18]. In addition, biologic activity has been proposed for both the soluble cadherin ectodomain and cytosolic intracellular domain [19, 20]. Soluble E-cadherin ectodomains were detected in increased amounts in sera from patients with several types of cancer and may promote cancer invasion and survival by blocking cell adhesion and stimulating growth factor receptor signaling [21–28]. For the released cadherin intracellular domain, it is likely rapidly degraded by the proteasome [16]. However, models that overexpress cadherin cytosolic domains have shown effects on gene transcription, including E-cadherin reversal of Kaiso-mediated gene repression by increased p120 nuclear localization [29] and N-cadherin-mediated loss of CRE-mediated gene transcription by increased cAMP response element-binding protein (CREB) binding protein degradation [30]. Cadherin-11 cleavage has not been previously reported in any mammalian system. Cadherin-11 cleavage by ADAMs was found to contribute to *Xenopus* cranial neurocrest migration, although the size of the cleavage fragments was not typical for ectodomain shedding followed by regulated intramembrane proteolysis [31].

As a recombinant cadherin-11 extracellular domain containing fusion protein was shown to stimulate a marked increase in human synovial fibroblast IL-6, chemokine, and metalloproteinase expression [3, 7], our objective was to determine if cadherin-11 ectodomain shedding occurs in the synovium and if a physiologic cleavage pathway in synovial fibroblasts releases cadherin-11 binding domain fragments. We found that shed soluble cadherin-11 extracellular domains (sCad11) are readily detected in joint synovial fluid and are increased in RA compared to osteoarthritis (OA) synovial fluid. These results provide direct evidence that a cadherin-11 cleavage pathway is active in the synovium. We showed that cadherin-11 cleavage can be stimulated by both calcium flux and TNF-α in human synovial fibroblasts, releasing first the cadherin-11 ectodomain and then the intracellular domain in a presenilin 1 (PSN-1) dependent manner. Unexpectedly, we found that a novel cell sheddase activity is responsible for cadherin-11 cleavage in primary human fibroblasts. Although ADAM10 mediated cadherin-11 cleavage in mouse embryonic fibroblasts and human lung cancer cells, inhibitor and short-interfering RNA (siRNA) studies indicate that the synovial fibroblast sheddase activity is ADAM- and metalloproteinase-independent. In summary, this study shows that cadherin-11 cleavage is active in the synovium and that cadherin-11 cleavage by synovial fibroblasts has unique characteristics when compared to other studied cell types.

## Methods

### Ethical approval

Human tissues for cell-line derivation and human synovial fluid were collected under the approval of the Institutional Review Board of Brigham and Women’s Hospital. As all specimens were obtained from discarded clinical specimens without identifying patient information, direct patient consent was not required for this study.

### Cells, media, and culture conditions

Human OA and RA synovial fibroblasts were isolated as previously described [32] from tissues discarded after synovectomy or joint replacement surgery and used experimentally between passages 5 and 10. Other cell lines were obtained as follows: ADAM-deficient mouse embryonic fibroblasts (MEFs) (Drs Carl Blobel and Paul Saftig), normal human lung fibroblasts (Lonza, Allendale, NJ, USA), and NCI-H460 lung cancer cells (ATCC, Manasses, VA, USA). The above cells were maintained in DMEM supplemented with 10% FBS (Gemini Bio-Products, West Sacramento, CA, USA), 2 m*M* L-glutamine, 100 units/ml penicillin, 100 μg/ml streptomycin, 50 μ*M* 2-mercaptoethanol (2-ME), and essential and nonessential amino acids (Life Technologies, Grand Island, NY, USA). Normal human skin fibroblasts were obtained from discarded human foreskin specimens that were first digested overnight at 4°C to remove the epithelium with a 10 mg/ml dispase II solution (Sigma-Aldrich, St. Louis, MO, USA) mixed 1:1 in RPMI 1640 medium containing 1% bovine serum albumin, 100 units/ml penicillin, 100 μg/ml streptomycin, 100 μg/ml gentamicin, and 0.25 μg/ml fungizone (Life Technologies). Fibroblasts were then isolated from the remaining dermal fragments by digestion at 37°C in DMEM containing 2 mg/ml type IV collagenase (Worthington Biochemical, Lakewood, NJ, USA), 0.8 mg/ml dispase (Roche Appliced Science, Indianapolis, IN, USA), and 0.1 mg/ml DNase I (Roche) and sequentially passaged in DMEM/F12 medium (Life Technologies) supplemented with 10% FBS, N-2-hydroxyethylpiperazine-N-2-ethane sulfonic acid (HEPES), L-glutamine, penicillin, streptomycin, and gentamicin. To examine cleavage, confluent cell cultures were first starved overnight in Opti-MEM medium (Life Technologies) before stimulation. Human synovial micromass cultures were generated as previously described [4].

### Antibodies and other reagents

The antibodies used are as follows: mouse IgG1 isotype control (MOPC-21, Bio X Cell); anti-cadherin-11 monoclonal antibody 23C6 and 3H10 [6]; anti-cadherin-11 monoclonal antibody 5B2H5 and polyclonal antibody #71-7600 (Life Technologies); anti-β-actin (AC-15, Sigma-Aldrich); anti-presenilin 1 (D39D1) and anti-presenilin 2 (Cell Signaling Technology, Danvers, MA, USA); anti-ADAM 10 (AB19026, EMD Millipore, Billerica, MA, USA). Cadherin-11-Fc and E-cadherin-Fc fusion proteins were generated as previously described [3, 33]. The other reagents used are as follows: ionomycin (Sigma-Aldrich); human TNF-α and platelet-derived growth factor (PDGF)-BB (R&D Systems, Minneapolis, MN, USA); L-685,458, lactacystin, batimastat, (TAPI-2), marimastat, and GM6001 (EMD Millipore). Culture media was concentrated using iCON™ 9kD concentrators (Thermo Scientific, Grand Island, NY, USA).

### siRNA transfection

Pooled siRNA against PSN1 and PSN2 and single siRNA sequence against ADAM10 (sc-41410C) were purchased from Santa Cruz Biotechnology. One control siRNA (also referred to as control 1 siRNA) was purchased from Thermo Scientific (On Target Pulse Non-targeting siRNA #1). The sequence for control 2 siRNA is 5′ CAACAAGAUGAAGAGCACCAAUU 3′ (synthesized by Thermo Scientific) [7]. Primary human fibroblasts were doubly transfected (at 0 and 48 hours) overnight with siRNA using the lipid reagent Dharmafect 3 (Thermo Scientific). Cells were rested for 24 hours and then serum-starved overnight in Opti-MEM before ionomycin stimulation. NCI-H460 cells were singly transfected with siRNA using the lipid reagent Dharmafect 1 (Thermo Scientific) for 24 hours and then serum-starved overnight in Opti-MEM before stimulation.

### Cell lysis, western blot, and immunoprecipitation

All cells were lysed with radioimmunoprecipitation assay (RIPA) buffer: 20 mM Tris pH 7.4, 150 mM NaCl, 0.5% deoxycholate, 1% Triton X-100, 0.1% sodium dodecyl sulfate (SDS), 1 mM EDTA, 1 mM sodium orthovanadate, 1 mM PMSF, and a mixture of protease inhibitors (Complete, EDTA-free, Roche Applied Science). Lysates were cleared of insoluble material by centrifugation. As noted, lysates were immunoprecipitated with the indicated antibodies using protein G sepharose beads (GE Healthcare Life Sciences, Pittsburgh, PA, USA). Samples were electrophoresed through polyacrylamide-SDS gels and then transferred to Immuno-Blot polyvinylidene fluoride (PVDF) membranes (Bio-Rad, Hercules, CA, USA). Membranes were blocked in phosphate-buffered saline containing 0.05% Tween and 5% bovine serum albumin and then incubated with antibodies against the target proteins. After washing, the membranes were incubated with horseradish peroxidase-conjugated anti-mouse IgG or anti-rabbit IgG antibodies (Jackson Immuno Research, West Grove, PA, USA) before detection with a chemiluminescent substrate. Full images for all cropped blots are provided in Additional file 1 and Additional file 2.

### Synovial fluid studies

For immunoprecipitation studies, synovial fluid (200 μl) was digested with 25 μg/ml hyaluronidase (Sigma Aldrich) for 15 minutes at room temperature. Prior to immunoprecipitation and western blot analysis, synovial fluid was pre-cleared with protein G-sepharose beads and human immunoglobulins and albumin were then removed using a high-affinity spin column (Thermo Scientific). To detect sCad11 by ELISA, high-binding 96-well plates (Costar, Tewksbury, MA, USA) were coated with anti-cadherin-11 monoclonal antibody 3H10 (4 μg/ml) and then blocked in HBS Ca (20 mM Hepes, 137 mM NaCl, 3 mM KCl, 1 mM CaCl, pH 7.4) with 1% bovine serum albumin. Undiluted synovial fluid was then incubated in the wells for 2 hours at room temperature. After washing, biotinylated anti-cadherin-11 antibody 23C6 (2 μg/ml) was added for 2 hours at room temperature. Binding of cadherin-11 extracellular fragments was detected by incubation with a streptavidin-alkaline phosphatase followed by colorimetric detection with *p*-nitrophenyl phosphate substrate.

### Statistical analysis

For pooled analyses, n refers to the number of independent experiments, with an experiment containing the results from one donor-derived fibroblast population. The number of unique donor-derived fibroblasts used is also indicated in these experiments. Often independent experiments were conducted more than once with a given fibroblast using different passages. All statistical analyses were calculated using GraphPad Prism software. All mean pixel density measurements from western analysis were calculated using Image J software.

## Results

**Fig. 1:**
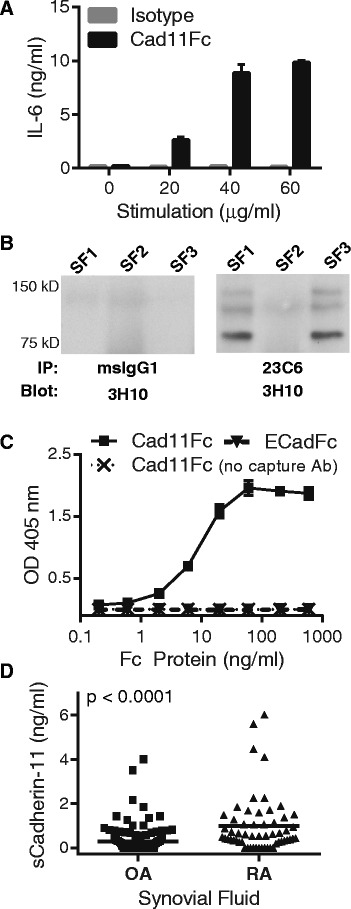
Evidence for cadherin-11 extracellular domain shedding in osteoarthritis (OA) and rheumatoid arthritis (RA) synovium. (**a**) Serum-starved RA synovial fibroblasts were incubated overnight with increasing concentrations of cad11Fc or isotype control antibody. IL-6 release was then measured by ELISA. Similar results were previously published and further characterized as discussed [3]. (**b**) Synovial fluid samples from OA patients were immunoprecipitated with a cadherin-11 extracellular domain antibody (23C6) followed by western blot analysis with a distinct cadherin-11 antibody (3H10). (**c**) Detection of the chimeric proteins cadherin-11-Fc or E-cadherin-Fc was measured using an ELISA developed to recognize the human cadherin-11 extracellular domain. The ELISA capture antibody was removed from the assay to serve as a negative control. The ELISA uses two anti-cadherin-11 monoclonal antibodies (23C6 and 3H10) recognizing distinct extracellular epitopes. (**d**) Soluble cadherin-11 fragments were detected by cadherin-11 extracellular domain specific ELISA in OA (n = 143, mean +/− standard deviation 0.28 +/− 0.56 ng/ml) and RA (n = 57, mean +/− standard deviation 0.99 +/− 1.3 ng/ml) synovial fluid patient samples. Cadherin-11 levels were significantly higher in RA synovial fluid (*P* <0.0001, two-tailed Student *t*-test)

We previously observed that a recombinant cadherin-11 extracellular domain Fc protein activated multiple signaling pathways in synovial fibroblasts, inducing expression of IL-6 and other mediators important in RA pathogenesis (see Fig. 1a, and previous studies [3, 7]). These studies suggest that soluble cleavage fragments containing cadherin-11 extracellular domain, if present in the synovium, may have biologic activity. Therefore, we determined if intact, soluble cadherin-11 extracellular domains (sCad11) could be isolated from patient synovial fluid specimens. We first demonstrated by immunoprecipitation that an approximately 75kD-band with a size consistent for sCad11 was detected in synovial fluid specimens (Fig. 1b). We then developed an ELISA to detect sCad11 from patient samples. In this assay, sCad11 is first captured with an extracellular cadherin11 epitope antibody and then detected with a separate biotinylated extracellular cadherin11 epitope antibody that does not interfere with epitope binding by the first antibody. This assay specifically detects cadherin-11 extracellular domains, but not ectodomains from another cadherin, E-cadherin (Fig. 1c). Using this assay, we determined the sCad11 levels in synovial fluid obtained from both OA and RA patients (Fig. 1d). Although sCad11 was detected in both, sCad11 levels were significantly increased in RA samples, likely reflecting the increased amount of synovial tissue present in RA. These results provide direct evidence supporting activity of a cadherin-11 cleavage pathway *in vivo*.

**Fig. 2:**
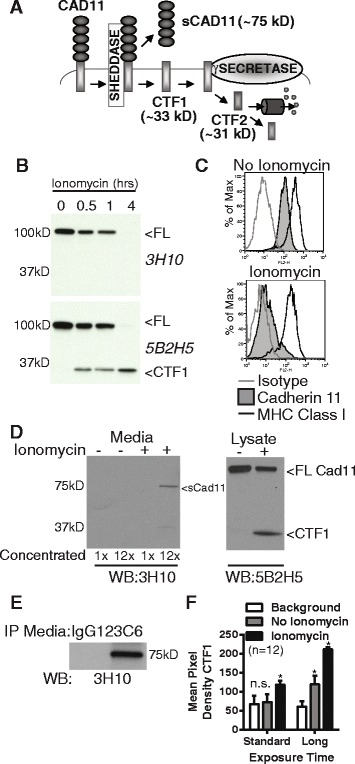
Cadherin-11 cleavage stimulation by ionomycin. (**a**) Proposed cadherin-11 cleavage model shows that cadherin-11 is first cleaved extracellularly at the plasma membrane by a cell sheddase, generating a C-terminal fragment 1 (CTF1) and an extracellular domain fragment (sCad11). Then CTF1 is cleaved near the intracellular plasma membrane, releasing C-terminal fragment 2 (CTF2) into the cytosol. CTF2 is likely rapidly degraded by the proteasome but may also effect gene transcription. (**b**) Lysates from RA synovial fibroblasts stimulated with 5 μM ionomycin as indicated were analyzed by western blot using antibodies directed against cadherin-11 extracellular (3H10) or intracellular (5B2H5) epitopes (FL, full length cadherin-11). (**c**) Surface staining for cadherin-11 (3H10), MHC class I (W632) or isotype control on RA synovial fibroblasts before or after 1 hour ionomycin stimulation was determined by flow cytometry (representative, 2 experiments). (**d**) Culture media and cell lysates harvested from RA synovial fibroblasts treated with or without ionomycin for 1 hour were analyzed by western blot for extracellular (3H10) and intracellular (5B2H5) cadherin-11 epitopes. Culture media was left unconcentrated or concentrated approximately 12-fold (representative 3 experiments). (**e**) Culture media from unstimulated cells was immunoprecipitated with anti-cadherin-11 antibody 23C6 or appropriate isotype control prior to western blot analysis (representative 3 experiments). (**f**) CTF1 levels in twelve sequential experiments were assayed by measuring band pixel intensity inunstimulated and ionomycin-stimulated cell lysates by western blot using standard and longer exposure times. (Pooled, 6 cells lines. Statistical comparison to background by t-test. n.s. = not significantly different; * p<0.001).

As cadherin-11 is expression is restricted to fibroblasts in the synovium, we then determined if a physiologic cadherin-11 cleavage pathway generating such fragments is active in these cells. As ectodomain shedding followed by regulated intramembrane proteolysis has been described for several other cadherins [20], we hypothesized that a sequential two-step cleavage pathway also occurs for cadherin-11 (Fig. 2a). In this model, a cell sheddase cleaves cadherin-11 near the extracellular membrane face to release a soluble N-terminal fragment containing the intact binding domain (sCad11) and a membrane-tethered fragment C-terminal fragment 1 (CTF1). CTF1 may then be processed by PSN in the γ-secretase complex to release the cadherin-11 intracellular domain into the cytosol, referred to as C-terminal fragment 2 (CTF2). CTF2 may then be further degraded by the proteasome.

Our initial experiments investigated if a cadherin-11 cleavage pathway was active in primary human synovial fibroblasts. As calcium flux is a well-described stimulus for ectodomain shedding, human RA synovial fibroblasts were treated with ionomycin to induce cadherin-11 cleavage. Ionomycin stimulation led to a time-dependent loss of full-length (FL) cadherin-11 from cell lysates as measured by western blot (Fig. 2b). Analysis with an anti-cadherin-11 antibody directed against an intracellular epitope (5B2H5) showed correlated accumulation of a cadherin-11 fragment whose molecular weight was consistent with that predicted for CTF1 (approximately 33 kD). This fragment was not detected by an anti-cadherin-11 antibody directed against an extracellular epitope (3H10). Loss of cell surface cadherin-11 after ionomycin treatment was confirmed by flow cytometry (Fig. 2c). Compared to untreated cells, ionomycin-stimulated synovial fibroblasts showed marked loss of cadherin-11 staining, even though the expression of another cell surface molecule, major histocompatibility complex (MHC) class I, was preserved. Consistent with a cleavage mechanism where cell surface expression is lost through ectodomain shedding, an extracellular cadherin-11 fragment was also detected in the synovial fibroblast culture media. Western blot analysis of concentrated culture media from ionomycin-stimulated synovial fibroblasts showed that the extracellular 3H10 antibody detected an approximately 75-kD species, the size of which is consistent with the intact cadherin-11 ectodomain (Fig. 2d). Cell lysates showed parallel intracellular CTF1 accumulation after ionomycin stimulation. In addition, immunoprecipitation of unstimulated culture media with one extracellular domain anti-cadherin-11 antibody (23C6) followed by western blot with another (3H10) also detected a 75kD species, indicating that a low level of constitutive cadherin-11 shedding is present in synovial fibroblasts (Fig. 2E). Similarly, although CTF1 was readily detected in ionomycin-stimulated cells, no statistically significant detection of CTF1 over background was seen in unstimulated cells by western blot analysis using standard exposure times (Fig. 2f, Additional file 3). However, with longer exposures, constitutive CTF1 generation was visualized. Cadherin-11 cleavage was not unique to RA synovial fibroblasts, as a similar intracellular cleavage fragment was also detected in OA fibroblasts after ionomycin stimulation (Additional file 4).

**Fig. 3:**
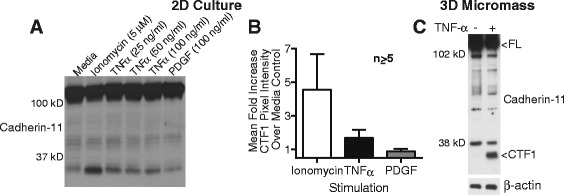
Stimulation of cadherin-11 cleavage by TNF-α. (**a**) Lysates from rheumatoid arthritis (RA) synovial fibroblasts left untreated or treated with ionomycin, TNF-α, or platelet-derived growth factor (PDGF)-BB for 2 hours were assayed for cadherin-11 cleavage using monoclonal antibody 5B2H5. (**b**) Mean fold increase in C-terminal fragment 1 (CTF1) pixel intensity for the indicated stimulation over unstimulated controls was calculated across several experiments using RA fibroblasts from different donors (fold change over media control (mean +/− standard error of mean): ionomycin (n = 7, 4 cell lines) 4.6 +/− 2.1; TNF-α 25 ng/ml (n = 74 cell lines) 1.7 +/− 0.17; PDGF-BB 100 ng/ml (n = 53 cell lines) 0.89 +/− 0.074). (**c**) Synovial fibroblast micromasses were cultured for 21 days, transferred to media containing 0.2 % serum for 24 hours, and then stimulated for 5 days with or without TNF-α (10 ng/ml). Cadherin-11 cleavage was then assessed using a rabbit polyclonal antibody against the cadherin-11 cytoplasmic domain (representative of at least three separate experiments with two cell lines). Equal protein loading was confirmed by β-actin staining

Although ionomycin potently stimulates cadherin-11 cleavage, we wished to determine if signals more physiologic to the inflamed RA synovium also might drive cleavage. We found that TNF-α treatment increased cadherin-11 cleavage in synovial fibroblast monolayers, although not as strongly as ionomycin (Fig. 3a-b). In comparison, platelet-derived growth factor (PDGF)-BB, a potent driver of cadherin-11 endocytosis and cell migration, did not induce any cadherin-11 cleavage. We then examined cadherin-11 cleavage in a three-dimensional synovial culture micromass to better mimic synovial architecture. In this system, we found that TNF-α also induced cleavage (Fig. 3c). These data, taken together, suggest that TNF-α likely provides an inflammatory signal that helps increase cadherin-11 cleavage in the RA synovium.

**Fig. 4:**
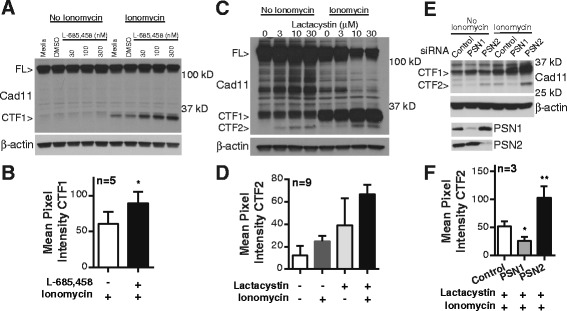
Involvement of γ-secretase in cadherin-11 cleavage. (**a**) Cell lysates from synovial fibroblasts incubated overnight increasing concentrations of the γ-secretase inhibitor L-685-458 and then stimulated as indicated were analyzed for cadherin-11 cleavage (representative 5 experiments with 4 cells lines, full-length cadherin-11 (FL); cadherin-11 (Cad11)). (**b**) CTF1 nean pixel intensity was calculated in lysates from ionomycin-stimulated synovial fibroblasts pretreated with or without L-685-458 (dose range 300–3000 nM, * p=0.0304 by paired t-test, error bars reflect standard error of the mean). (**c**) Cell lysates from RA synovial fibroblasts incubated overnight with increasing concentrations of the proteosome inhibitor lactacystin and then stimulated as indicated were analyzed for cadherin-11 cleavage (representative of 9 experiments with 5 cells lines). (**d**) CTF2 mean pixel intensity was calculated in lysates from synovial fibroblast treated with or without ionomycin (5 μM) and lactacystin (3–10 μM) as indicated (p<0.0001 by one-way ANOVA, error bars reflect standard error of the mean). (**e**) RA synovial fibroblasts were transfected with the indicated siRNAs, incubated overnight with 10 μM lactacystin, and then were analyzed for cadherin-11 cleavage in the presence or absence of ionomycin (representative 3 experiments with 3 cell lines). Silencing was confirmed by western blot. β-actin levels were used fro protein loading control. (**f**) Mean pixel intensity was calculated for CTF2 bands in siRNA-transfected RA synovial fibroblasts pretreated with 10 μM lactacystin and then stimulated with ionomycin (* p=0.0045, paired t-test; **p=0.0136 paired t-test, n=3, error bars reflect standard deviation of the mean).

Having identified a putative post-cleavage cadherin-11 intracellular fragment with a molecular size consistent for CTF1, proteasome and γ-secretase activity was then targeted to confirm its identity by showing this fragment undergoes regulated intramembrane proteolysis to generate CTF2. First, synovial fibroblasts were treated with the γ-secretase inhibitor L-685,458 to reduce cleavage of CTF1 to CTF2. Inhibitor-treated cells showed a dose-dependent increase in CTF1 after ionomycin stimulation (Fig. 4a-b), suggesting that CTF1 is further processed by the γ-secretase complex, as predicted in our cleavage model (Fig. 2a).

Next, as CTF2 is generally rapidly degraded by the proteasome, synovial fibroblasts were treated with the proteasome inhibitor lactacystin to enhance CTF2 detection. Lactacystin-treated cells showed a dose-dependent accumulation of a low molecular fragment with a size consistent for CTF2 (Fig. 4c-d). In this experiment, CTF1 and CTF2 were detected in both unstimulated and ionomycin-stimulated fibroblasts. Detection of CTF2 requires long exposure times. As shown earlier (Fig. 2f), long exposure times also detect a level of constitutive cleavage in unstimulated cells.

Finally, the catalytic component of γ-secretase, the aspartyl protease PSN, was silenced using siRNAs directed against the two homologous proteins, PSN1 and PSN2 (Fig. 4e-f). PSN1-silenced cells showed impaired ability to generate CTF2, strongly pointing to PSN1 as a critical mediator of cadherin-11 intramembrane cleavage. In contrast, PSN2 silencing increased CTF2 accumulation, with CTF2 detected even without stimulation (Fig. 4e-f), indicating PSN2 does not have a catalytic role in generating cadherin-11 CTF2. It is possible that absence of PSN2 increases PSN1-complex interactions with cadherin-11, promoting CTF2 generation. Taken together, these experiments demonstrate that γ-secretase mediates cadherin-11 cleavage, confirming cadherin-11 is a substrate for regulated intramembrane proteolysis in synovial fibroblasts.

After finding that cadherin-11 is cleaved by γ-secretase, we sought to identify which protease mediates the first cleavage step, generation of sCad11 and CTF1 from the full-length protein. The most likely candidate was an ADAM family member. ADAMs are the dominant cell sheddases in regulated intramembrane proteolysis for multiple proteins on various cell types [34, 35]. ADAM10, in particular, has been reported to cleave E-cadherin, N-cadherin, and VE-cadherin [18, 22, 29, 36–38] while ADAM15 may also contribute to E-cadherin cleavage [28]. Several ADAMs, including ADAM10, are expressed on synovial fibroblasts [39–41], suggesting ADAMs are candidates to mediate cadherin-11 cleavage.

**Fig. 5:**
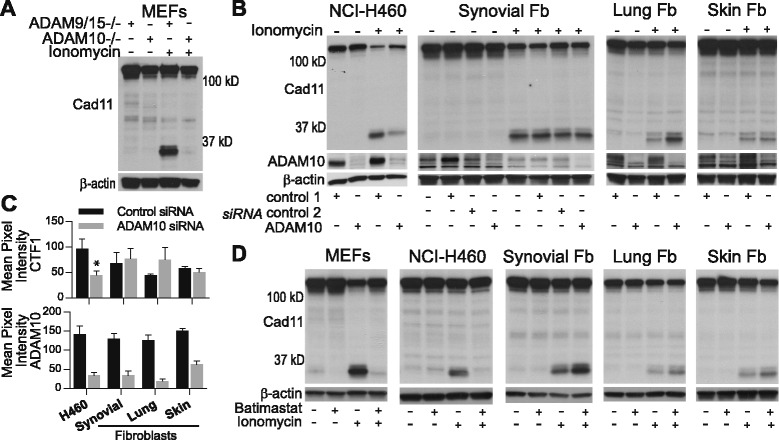
Comparison of sheddase activity in mouse embryonic fibroblasts (MEFs), NCI-H460, and primary human fibroblasts. (**a**) Lysates from MEFs genetically deficient in a disintegrin and metalloproteinase (ADAM10 −/−) or (**b**) from NCI-H460, synovial fibroblasts, lung fibroblasts, or skin fibroblasts transfected with control or ADAM10 siRNA were analyzed for cadherin-11 cleavage in the presence or absence of ionomycin (representative of at least three experiments, fibroblasts (Fb). ADAM10 siRNA silencing was confirmed by western blot and β-actin levels confirmed equal protein loading. (**c**) C-terminal fragment 1 (CTF1) and ADAM10 expression was measured by calculating the mean pixel intensity of CTF1 and ADAM10 bands across several experiments in control and ADAM10 siRNA treated cells for the indicated cell types (H460, n = 3; ^*^ *P* = 0.049 by paired *t*-test; lung and skin fibroblasts, n = 3; synovial fibroblasts, n = 6, five separate rheumatoid arthritis (RA) lines; error bars reflect standard error of mean). ADAM10 silencing efficiency (mean+/−standard deviation): H460 cells, 77.0 + 5.67%; synovial fibroblasts, 70.2+/−25.5%; lung fibroblast, 85.0+/−8.04%; skin fibroblasts, 58.8+/−7.77%. (**d**) Cell lysates from indicated cells treated overnight with the metalloproteinase inhibitor batimastat (10 μM) or appropriate dimethyl sulfoxide (DMSO) vehicle control were assayed for cadherin-11 cleavage in the presence and absence of ionomycin stimulation (representative of at least three experiments per cell type). Equal protein loading was confirmed by β-actin staining

To examine if ADAM proteins cleave cadherin-11, we used MEFs genetically deficient in both ADAM9 and ADAM15 or ADAM10 alone. Compared to ADAM9/ADAM15-deficient cells, generation of cadherin-11 CTF1 after ionomycin was completely absent in ADAM10-deficient cells (Fig. 5a), implicating ADAM10 as a sheddase for cadherin-11 cleavage. We then used siRNA to silence ADAM10 in human synovial fibroblasts. Surprisingly, despite efficient ADAM10 silencing, the absence of ADAM10 did not block cadherin-11 cleavage in synovial fibroblasts (Fig. 5b-c), and additional silencing of ADAM9, ADAM12, and ADAM15 also had no effect (data not shown). We then tested the effect of ADAM10 silencing in several additional human cell types. ADAM10 silencing in the lung cancer cells H460 inhibited generation of CTF1, but had no effect in two additional primary human fibroblast lines derived from skin and lung, respectively (Fig. 5b-c). These results suggested that although ADAM10 contributes to cadherin-11 cleavage in MEFs and human epithelial carcinoma, it does not in primary human fibroblasts obtained from various tissue sites.

As the majority of cell sheddase activity is reported to be mediated by metalloproteinases (for example, ADAMs and matrix metalloproteinases (MMPs)), we decided to use chemical inhibitors to examine if cadherin-11 cleavage is mediated by metalloproteinase activity (Fig. 5d). MEFs, H460 cells, synovial fibroblasts, lung fibroblasts, and skin fibroblasts were pretreated with the broad spectrum metalloproteinase inhibitor batimastat before ionomycin stimulation. As expected, CTF1 generation was completely inhibited by batimastat in both MEFs and H460 cells. Remarkably, batimastat did not block cadherin-11 cleavage in primary synovial, lung, or skin fibroblasts, even at a dose 100-fold higher than the dose needed to inhibit cleavage in H460 cells (Additional file 5). Additional metalloproteinase inhibitors (TAPI-2, marimastat, and GM6001) also did not block cleavage in synovial fibroblasts (Additional file 6), pointing to an unexpected, non-ADAM sheddase mediating cadherin-11 cleavage in primary human fibroblasts. Although the identity of the fibroblast sheddase remains to be determined, these results highlight that shedding of a cell surface protein may occur by very different mechanisms in distinct cell types.

## Discussion

We previously demonstrated that a recombinant soluble form of the extracellular cadherin-11 binding domain (cadherin-11-Fc) was highly active biologically and stimulated synovial fibroblasts to produce chemokines, cytokines, and MMPs in synergy with inflammatory cytokines, pointing to an important role for cadherin-11 signaling in RA disease pathogenesis [3, 7]. Although cadherin signaling may be induced through formation of new cell-to-cell contacts [11], it is also possible that soluble cadherin-11 extracellular domains shed from synovial fibroblasts may also contribute to those interactions. Cadherin-11 shedding from synovial fibroblasts seemed likely, given that protein ectodomain shedding has been described for several cadherins in other cell types [17, 18, 29, 30, 36]. In this study, we detected shed cadherin-11 ectodomains in joint synovial fluid, directly demonstrating that a cadherin-11 cleavage pathway is active in the synovium. We then showed that cadherin-11 undergoes protein ectodomain shedding followed by PSN-dependent regulated intramembrane proteolysis in synovial fibroblasts and discovered several novel features of that cleavage.

First, we showed that after ectodomain shedding, cadherin-11 is processed by the γ-secretase complex (Fig. 4). This complex is composed of four integral membrane proteins in 1:1:1:1 stoichiometry: presenilin, nicastrin, anterior-pharynx defective-1 (APH-1), and presenilin enhancer-2. Although enzymatic activity is mediated by PSN, the other components contribute to complex stability and substrate recognition [16, 42]. The PSN family contains two homologous proteins, PSN1 and PSN2, with approximately 67% amino acid identity. In addition, APH-1 also has two homologues, one of which has two splice variants. Therefore, up to six different γ-secretase forms may exist within a cell. This study contributes to emerging evidence showing that these distinct γ-secretase complexes may have unique substrate specificities and cellular locations. We demonstrated that PSN1, not PSN2, mediates cadherin-11 CTF1 to CTF2 processing. In fact, as PSN2 silencing increased CTF2 cleavage, PSN2 may act to inhibit PSN1 cleavage, possibly by binding to a subset of cadherin complexes. Whether PSN1-dependent cleavage is unique to cadherin-11 in synovial fibroblasts or is a more general feature of cadherin cleavage remains to be determined.

Ectodomain shedding is the rate-limiting step in regulated intramembrane proteolysis, and the dominant cell sheddases are ADAM family members [15, 35]. Several ADAMs, especially ADAM 10, are known to cleave other cadherins [18, 22, 29, 36–38]. In this study, we found that ADAM 10 can cleave cadherin-11 on MEFs and a lung cancer cell line, but show it does not cleave cadherin-11 on several types of primary human fibroblasts, despite detectable ADAM10 expression in these cells. In fact, using several chemical inhibitors, we found that the sheddase is likely not a metalloproteinase, excluding a role in cleavage for other ADAMs or MMPs. The identity of the cadherin-11 sheddase on human fibroblasts is currently not known. One possible candidate is a member of the BACE family of aspartyl proteases, another important sheddase family [15, 43]. However, BACE proteins currently have a very limited number of identified substrates, and no activity against cadherins has been reported. Another possibility includes the serine proteases kallikrein and plasmin, both of which release the E-cadherin extracellular domain in recombinant protein systems [24, 26]. However, these studies did not show shedding in a cell system or link this shedding to subsequent processing by γ-secretase.

Further investigations are needed to identify the cadherin-11 sheddase activity and understand why ADAM10 does not mediate cleavage despite detectable expression on synovial fibroblasts. However, some predictions about the cleavage region can be made by examining the ionomycin-induced metalloproteinase cleavage sites identified for E-cadherin [17] and N-cadherin [37]. For both of these cadherins, cleavage occurs very proximally, within 10 amino acid of the transmembrane domain. For E-cadherin, cleavage occurs between Proline^700^ and Valine^701^, while for N-cadherin, cleavage involves Isoleucine^715^. Sequence alignment of these regions with cadherin-11 shows the likely area of cleavage to generate cadherin-11 ectodomains (Additional file 7). We do not know whether or not metalloproteinase-mediated cleavage in H460 cells shares the same cleavage site as non-metalloproteinase-mediated cleavage in primary fibroblasts. However, given the similar size of shed cadherin-11 ectomains and CTF1 in these two cell types, we would predict that the cleavage sites, if not identical, would be very close to each other.

Although stimulating fibroblasts *in vitro* with ionomycin, a potent inducer of calcium flux, is helpful in studying cleavage mechanisms, it remains an artificial system. As the major mechanism of cadherin turnover is through the endocytic pathway, we wished to determine if cadherin-11 cleavage occurs *in vivo* in the synovium. Using both western blot and ELISA analyses, we detected the soluble extracellular cadherin-11 domain in synovial fluid specimens, providing strong evidence for an active cadherin-11 cleavage pathway in the synovium (Fig. 1). Furthermore, the amount of cleavage fragments was significantly enhanced in RA synovial fluid compared to OA, suggesting that cleavage may correlate with the amount of synovial tissue or inflammatory signals. Indeed, we found that the inflammatory cytokine, TNF-α, also induced cleavage, both in standard monolayer cultures and in a three-dimensional culture system that more closely mimics synovial lining structure.

Although cadherin-11 cleavage occurs in the synovium, the functional consequences of that cleavage are not known. One possibility is that cadherin-11 cleavage disrupts cell-to-cell contacts, potentially altering the adhesive state of synovial fibroblasts. Indeed, stimulating E-cadherin cleavage disrupts cell contact in A431 epidermoid carcinoma cells [17], while inhibiting ADAM protease activity in MEFs and human umbilical vein endothelial cells increased surface N-cadherin and VE-cadherin levels, respectively [18, 36]. However, how much or when cadherin-11 proteolysis contributes to cadherin-11 turnover is uncertain, as the major pathway for cadherin contact remodeling is generally accepted to occur through endocytosis [13].

Nevertheless, a strong case can be made that the shed cadherin extracellular domains may have biologic activity [19, 21]. In tumor studies, cadherin extracellular domains promote cell migration and invasion likely by inhibiting cadherin cell adhesion interactions [24–27]. However, soluble forms of E-cadherin have also been shown to directly bind to and stimulate the growth factor receptors, epidermal growth factor receptor (EGFR), Her2, and Her3 [22, 23, 28], activating cell signaling pathways. Our work showed that a recombinant chimeric molecule containing the cadherin-11 extracellular domain stimulated pro-inflammatory mediator production in synovial fibroblasts [3, 7], suggesting that shed cadherin-11 promotes synovial activation in RA. However, *in vitro* activation of synovial fibroblasts requires substantially higher concentrations of cad11Fc compared to the concentrations of shed cadherin-11 detected in synovial fluid. On the other hand, synovial fluid levels are unlikely to reflect the true local concentration of shed cadherin-11 in the synovial lining. Further investigation to determine the biologic function of shed cadherin-11 in the synovium may need to model the effect of shed cadherin-11 in a three dimensional system that mimics synovial lining architecture.

Another possibility is that the intracellular domain fragment released into the cytosol, CTF-2, also may have biologic activity. CTF2 retains the ability to bind β-catenin and p120 catenin, both of which can localize to the nucleus to affect gene transcription [10]. In fact, E-cadherin CTF2 overexpression in MDCK cells resulted in increased transport of p120 catenin to the nucleus, allowing p120 to relieve Kaiso-mediated gene repression [29]. In addition, N-cadherin CTF2 overexpression in mouse L cells increased degradation of CREB-binding protein by the proteosome, reducing CRE-mediated gene transcription [30]. Atlhough it is intriguing to consider CTF2 as a signaling molecule, rapid proteosomal degradation of CTF2 in the absence of an overexpression system makes it more difficult to assign biologic activity to cadherin CTF2 fragments. It remains to be determined if cadherin-11 CTF2 has any functional activity in synovial fibroblasts.

## Conclusion

We previously found that a cadherin-11 extracellular domain fusion protein stimulated synovial fibroblasts to produce inflammatory and degradative mediators important in RA pathogenesis. In this study, we present evidence that cadherin-11 extracellular domains are shed from synovial fibroblasts by a novel, non-metalloproteinase sheddase and then further cleaved by regulated intramembrane proteolysis to release the cytoplasmic domain. Furthermore, shed cadherin-11 extracellular domains were readily isolated from patient synovial fluid and were significantly enriched in RA compared to OA samples, indicating that this cleavage pathway is active in the synovium. We propose that shed cadherin-11 may help promote RA pathogenesis by acting in synergy with inflammatory cytokines to promote synovial fibroblast activation.

## Additional files

Additional file 1: Figure. S1. Complete gels for the cropped images in Fig. 1b, Fig. 2e, Fig. 3c, and Fig. 4a-c are provided in this file. Additional file 2: Figure. S2. Complete gels for the cropped images in Fig. 5 are provided in this file. Additional file 3: Figure. S3. Constitutive and induced C-terminal fragment 1 (CTF1) generation in rheumatoid arthritis (RA) synovial fibroblasts. Cell lysates from RA synovial fibroblasts stimulated with or without 5 μM ionomycin for one hour were analyzed for cadherin-11 cleavage by western blot. Two exposure times of the western blot are shown- standard and long. Representative figure for the pooled data shown in Fig. 2f. Additional file 4: Figure. S4. Cadherin-11 cleavage in osteoarthritis (OA) synovial fibroblasts. Cell lysates from an OA synovial fibroblast line stimulated with or without 5 μM ionomycin for one hour were analyzed for cadherin-11 cleavage by western blot. Additional file 5: Figure. S5. Effect of increasing doses of the metalloproteinase inhibitor batimastat in NCI-H460 cells and synovial fibroblasts. (**a**) NCI-H460 cells or (**b**) rheumatoid arthritis (RA) synovial fibroblasts were treated overnight without or with increasing concentrations of batimastat and then left unstimulated or stimulated with 5 μM ionomycin for one hour before lysis. Cell lysates were analyzed for cadherin-11 cleavage by western blot. Equal protein loading was confirmed by β-actin staining. Additional file 6: Figure. S6. Effect of several metalloproteinase inhibitors in mouse embryonic fibroblasts (MEFs) or synovial fibroblasts. (**a**) MEFs or rheumatoid arthritis (RA) synovial fibroblasts were left untreated or treated overnight with 30 μM batimastat, 100 μM TNF-α protease inhibitor 2 (TAPI-2), or dimethyl sulfoxide (DMSO) vehicle control. Ionomycin (5 μM) or vehicle control was then added for one hour before cell lysis. Cell lysates were analyzed for cadherin-11 cleavage by western blot. Equal protein loading was confirmed by β-actin staining. (**b**) RA synovial fibroblasts were left untreated or pretreated with marimastat, GM6001, or DMSO vehicle control before addition of ionomcyin or vehicle control. Cell lysates were then analyzed for cadherin-11 cleavage by western blot. Additional file 7: Figure. S7. Comparison of cadherin-11 sequence to known metalloproteinase cleavage sites in other cadherins. E-cadherin, N-cadherin, and cadherin-11 extracellular domain amino acid sequences just proximal to the transmembrane domain are aligned. Known amino acids acid involved in metalloproteinase-mediated ectodomain shedding are indicated by bold, underlined typeface.

## Abbreviations

ADAM: a disintegrin and metalloproteinase
BACE1: beta-secretase 1
CTF1: C-terminal fragment 1
CTF2: C-terminal fragment 2
DMEM: Dulbecco’s modified Eagle’s medium
DMSO: dimethyl sulfoxide
ELISA: enzyme-linked immunosorbent assay
FBS: fetal bovine serum
I-CLiPs: intramembrane-cleaving proteases
IL: interleukin
MEF: mouse embryonic fibroblast
MHC: major histocompatibility complex
MMP: matrix metalloproteinase
OA: osteoarthritis
PDGF: platelet-derived growth factor
PSN1: presenilin 1
PSN2: presenilin 2
RA: rheumatoid arthritis
RPMI: Roswell Park Memorial Institute
sCad11: soluble cadherin-11
siRNA: short-interfering RNA
TNF-α: tumor necrosis factor alpha
