# Supplementary figures and images for: Evidence for cadherin-11 cleavage in the synovium and partial characterization of its mechanism
Source: Arthritis Res Ther. 2015 May 15;17(1):126. doi: 10.1186/s13075-015-0647-9 (PMC4449585; doi:10.1186/s13075-015-0647-9)

**Fig. 1B**

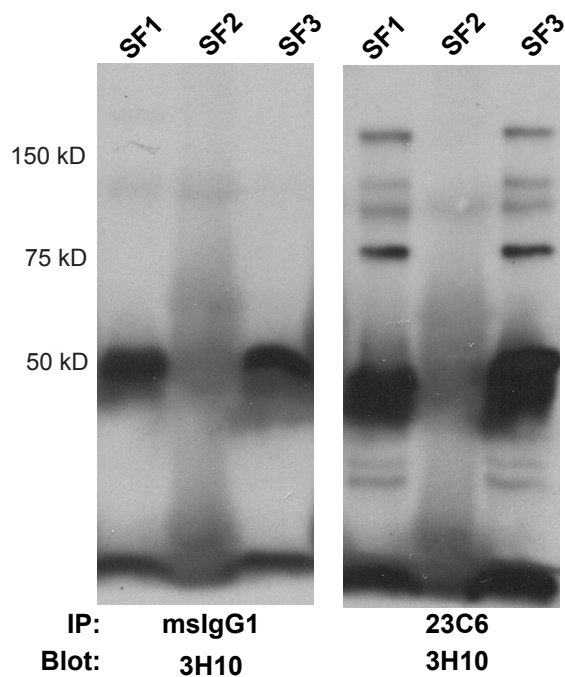

**Fig. 2E**

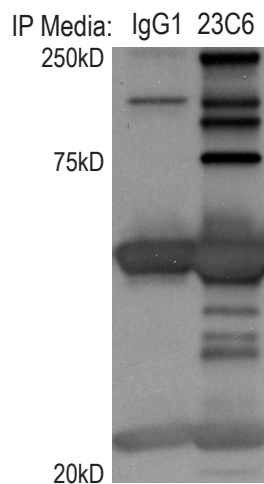

**Fig. 3C**

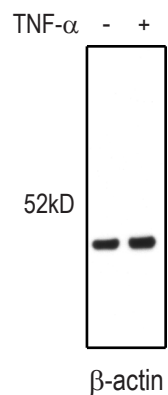

**Fig. 4A**

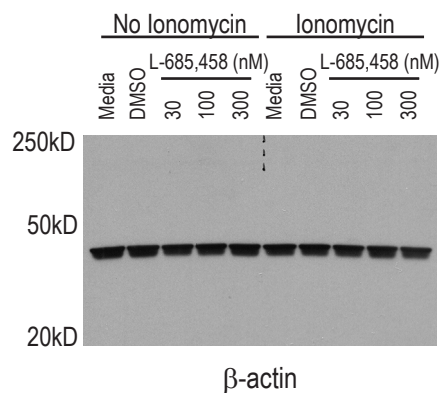

**Fig. 4B**

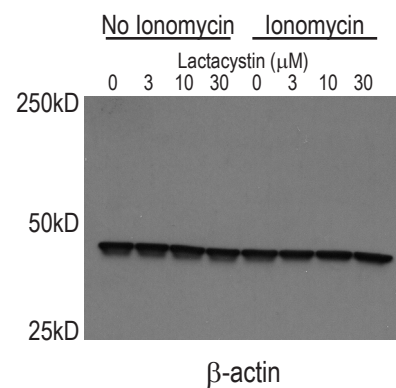

**Fig. 4C**

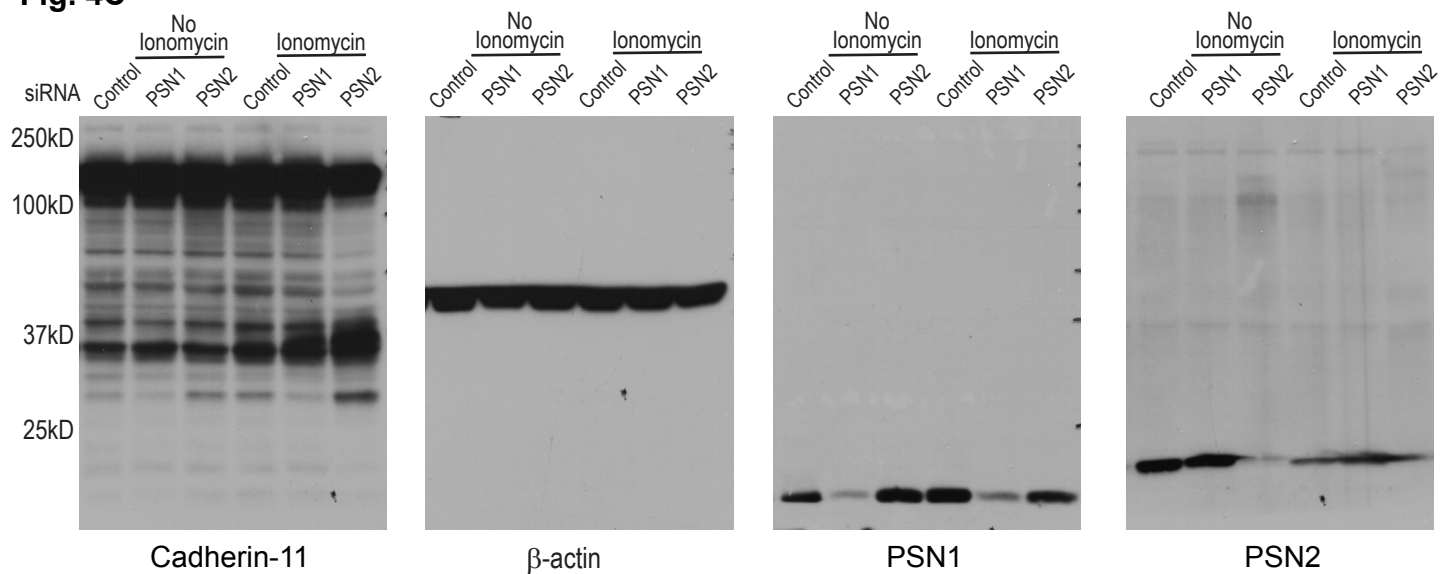

Supplement: Additional file 1: Figure. S1. — Complete gels for the cropped images in Fig. 1b, Fig. 2e, Fig. 3c, and Fig. 4a-c are provided in this file. [file 13075_2015_647_MOESM1_ESM.pdf]

**Fig. 5A**

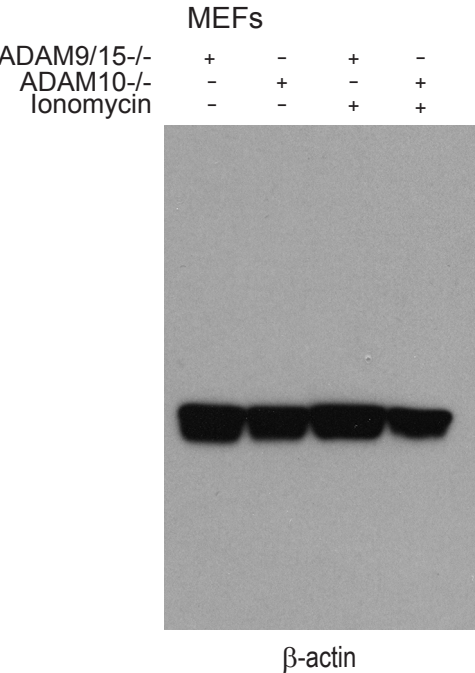

**Fig. 5B**

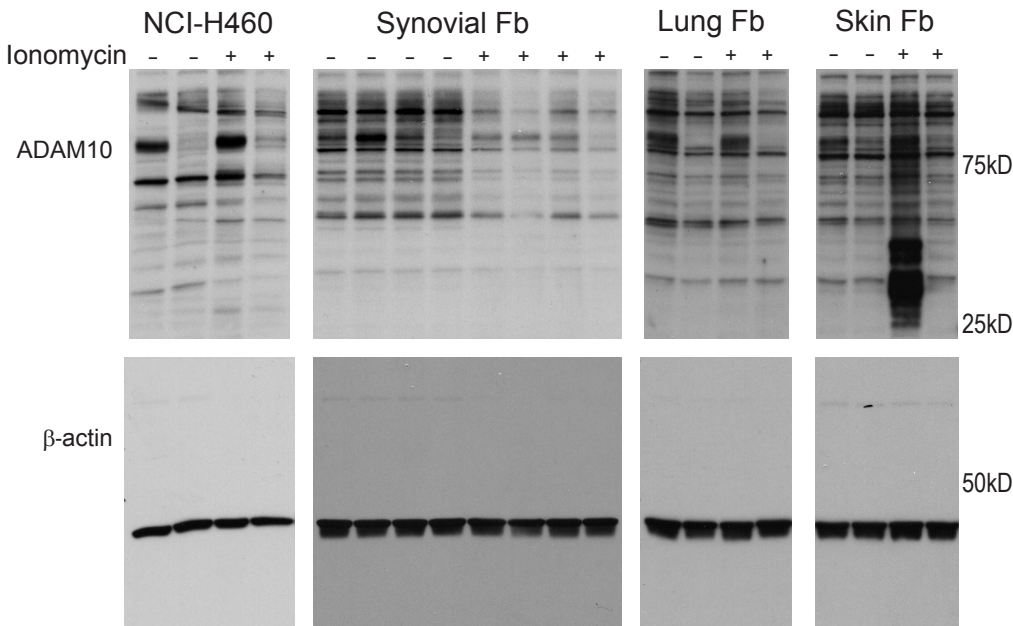

**Fig. 5C**

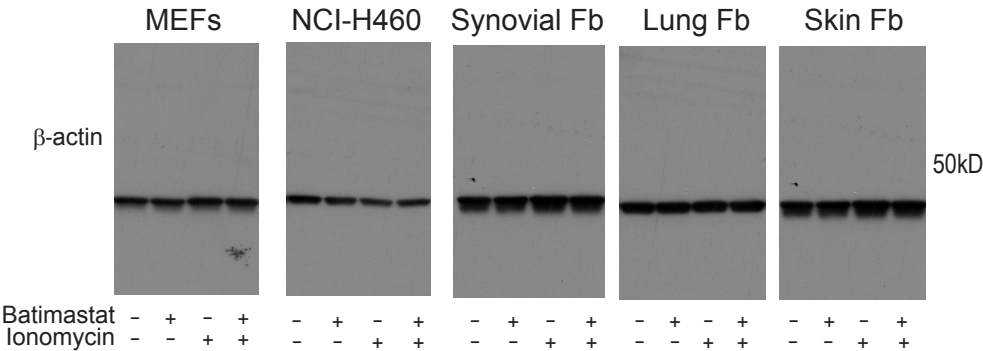

Supplement: Additional file 2: Figure. S2. — Complete gels for the cropped images in Fig. 5 are provided in this file. [file 13075_2015_647_MOESM2_ESM.pdf]

**Ionomycin**

**-**

**+**

**-**

**+**

FL cadherin-11 >

100 kD

100 kD

37kD

37kD

CTF1 >

Standard  
Exposure

Long  
Exposure

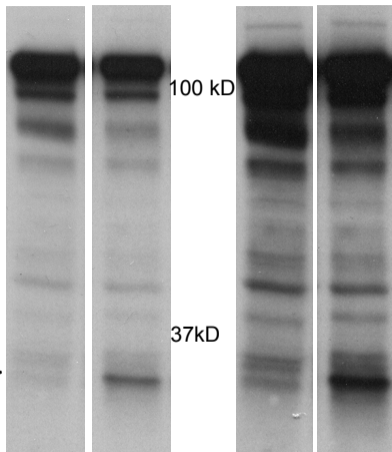

Supplement: Additional file 3: Figure. S3. — Constitutive and induced C-terminal fragment 1 (CTF1) generation in rheumatoid arthritis (RA) synovial fibroblasts. Cell lysates from RA synovial fibroblasts stimulated with or without 5 μM ionomycin for one hour were analyzed for cadherin-11 cleavage by western blot. Two exposure times of the western blot are shown- standard and long. Representative figure for the pooled data shown in Fig. 2f. [file 13075_2015_647_MOESM3_ESM.pdf]

lonomycin

-

+

100 kD

37 kD

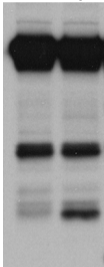

OA4

Supplement: Additional file 4: Figure. S4. — Cadherin-11 cleavage in osteoarthritis (OA) synovial fibroblasts. Cell lysates from an OA synovial fibroblast line stimulated with or without 5 μM ionomycin for one hour were analyzed for cadherin-11 cleavage by western blot. [file 13075_2015_647_MOESM4_ESM.pdf]

**A**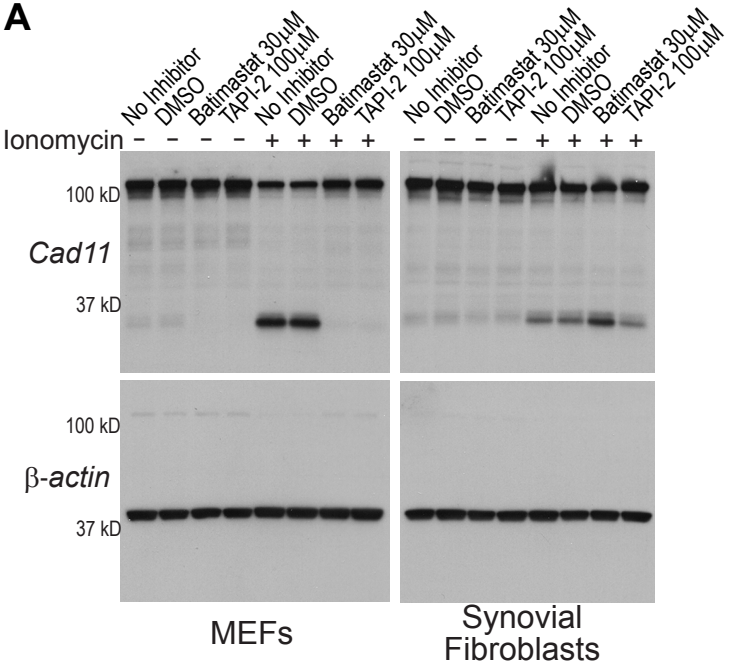**B**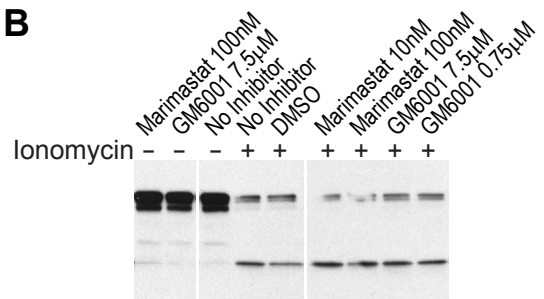

Supplement: Additional file 6: Figure. S6. — Effect of several metalloproteinase inhibitors in mouse embryonic fibroblasts (MEFs) or synovial fibroblasts. (a) MEFs or rheumatoid arthritis (RA) synovial fibroblasts were left untreated or treated overnight with 30 μM batimastat, 100 μM TNF-α protease inhibitor 2 (TAPI-2), or dimethyl sulfoxide (DMSO) vehicle control. Ionomycin (5 μM) or vehicle control was then added for one hour before cell lysis. Cell lysates were analyzed for cadherin-11 cleavage by western blot. Equal protein loading was confirmed by β-actin staining. (b) RA synovial fibroblasts were left untreated or pretreated with marimastat, GM6001, or DMSO vehicle control before addition of ionomcyin or vehicle control. Cell lysates were then analyzed for cadherin-11 cleavage by western blot. [file 13075_2015_647_MOESM6_ESM.pdf]
