# Supplementary material for: Evidence for cadherin-11 cleavage in the synovium and partial characterization of its mechanism
Source: Arthritis Res Ther. 2015 May 15;17(1):126. doi: 10.1186/s13075-015-0647-9 (PMC4449585; doi:10.1186/s13075-015-0647-9)
Supplement: Additional file 5: Figure. S5. — Effect of increasing doses of the metalloproteinase inhibitor batimastat in NCI-H460 cells and synovial fibroblasts. (a) NCI-H460 cells or (b) rheumatoid arthritis (RA) synovial fibroblasts were treated overnight without or with increasing concentrations of batimastat and then left unstimulated or stimulated with 5 μM ionomycin for one hour before lysis. Cell lysates were analyzed for cadherin-11 cleavage by western blot. Equal protein loading was confirmed by β-actin staining. [file 13075_2015_647_MOESM5_ESM.pdf]

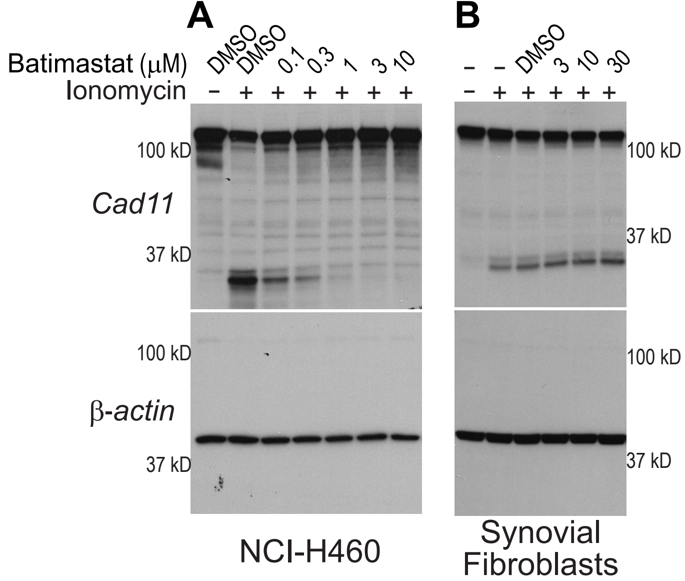

A
B
Batimastat (μM)
DMSO
DMSO
0.1
0.3
1
3
10
–
–
DMSO
3
10
30
Ionomycin
–
+
+
+
+
+
+
–
+
+
+
+
+
100 kD
Cad11
37 kD
100 kD
37 kD
100 kD
β-actin
37 kD
100 kD
37 kD
NCI-H460
Synovial Fibroblasts
