# Supplementary material for: Evidence for cadherin-11 cleavage in the synovium and partial characterization of its mechanism
Source: Arthritis Res Ther. 2015 May 15;17(1):126. doi: 10.1186/s13075-015-0647-9 (PMC4449585; doi:10.1186/s13075-015-0647-9)
Supplement: Additional file 7: Figure. S7. — Comparison of cadherin-11 sequence to known metalloproteinase cleavage sites in other cadherins. E-cadherin, N-cadherin, and cadherin-11 extracellular domain amino acid sequences just proximal to the transmembrane domain are aligned. Known amino acids acid involved in metalloproteinase-mediated ectodomain shedding are indicated by bold, underlined typeface. [file 13075_2015_647_MOESM7_ESM.pdf]

681 700-701 711
E-cadherin ...LEVSVCDCEGAAGVCRKAQ**PV**EAGLQIPAIL.....

691 715 726
N-cadherin ...RVKVCQCDSNGGDCTDVDR**I**VGAGLGTGAII.....

591 621
Cadherin-11...KVCGCDVNGALLSCNAEAYILNAGLSTGALI.....

EXTRACELLULAR | TRANSMEMBRANE
